# Dataset of long term variation in species occurrence and abundance of tintinnid assemblages in Jiaozhou Bay, China

**DOI:** 10.1016/j.dib.2018.06.010

**Published:** 2018-06-15

**Authors:** Meiping Feng, Chaofeng Wang, Wuchang Zhang, Guangtao Zhang, Henglong Xu, Yuan Zhao, Tian Xiao, Chunsheng Wang, Weiding Wang, Yuanxin Bi, Jun Liang

**Affiliations:** a CAS Key Laboratory of Marine Ecology and Environmental Sciences, Institute of Oceanology, Chinese Academy of Sciences, Qingdao 266071, China; b Laboratory for Marine Ecology and Environmental Science, Qingdao National Laboratory for Marine Science and Technology, Qingdao 266071, China; c Marine and Fisheries Research Institute, Zhejiang Ocean University, Zhoushan 316000, China; d Jiaozhou Bay Marine Ecosystem Research Site, Institute of Oceanology, Chinese Academy of Sciences, Qingdao 266071, China; e Key Laboratory of Marine Ecosystem and Biogeochemistry, Second Institute of Oceanography, State Oceanic Administration, Hangzhou 310012, China; f Department of Marine Ecology, Ocean University of China, Qingdao 266003, China

## Abstract

This article contains supportive data related to a research article entitled “Annual variation of species richness and lorica oral diameter characteristics of tintinnids in a semi-enclosed bay of western Pacific” (Feng et al., 2018) [1]. This article describes long term data of tintinnid assemblages in Jiaozhou Bay, Yellow sea, a semi-enclosed basin ecosystem of western Pacific, from May 2003 to December 2012. We sum up the whole dataset for each year showing tintinnid species occurrence and abundance at each site by date, as well as the photographic documentation of each tintinnid species. Further interpretation and discussion can be found in recently published by Feng et al. in Estuarine, Coastal and Shelf Science at Science.

**Specifications Table**

**Table:** 

Subject area	*Biology*
More specific subject area	*Marine ecology, microzooplankton, ciliates*
Type of data	*Table, image (microscopy)*
How data was acquired	*Survey, Microscope*
Data format	*Raw and processed data*
Experimental factors	*Field sampling at 4 sites monthly during a ten-year cycle*
Experimental features	*Observed and counted under microscope using method of Utermöhl (1958) after filtered through a net (mesh size 20 μm)*
Data source location	*Jiaozhou Bay, China, 35.98-36.16°N, 120.25-120.43°E*
Data accessibility	*data is with this article*
Related research article	[1]*Feng MP, Wang CF, Zhang WC, et al. Annual variation of species richness and lorica oral diameter characteristics of tintinnids in a semi-enclosed bay of western Pacific. Estuarine, Coastal and Shelf Science. 2018 207:164–174.*

**Value of the data**

● This dataset contains raw and processed data of tintinnid assemblages, one of the important microzooplankton groups, at 4 sampling sites during a ten-year cycle in a semi-enclosed Bay in western Pacific.
● The variation of plankton groups as well as phenology and be explored and analyzed using such statistical methods as analysis of variance, regression, factor analysis, cluster analysis, or structural equation modeling.
● New collaborations about copepods, Chl *a*, and other zooplankton groups, and microbial food loop are welcome.

## 1 Experimental design, materials and methods

Tintinnid samples were collected aboard R/V ‘Kejiao No.1’ in Jiaozhou Bay, Yellow sea, in the temperate western Pacific. Samplings were conducted at 4 sites (St. A5, C3, D7, and D8) once a month from May 2003 to December 2012. A 30 L surface water was collected at each site by a large volume water sampler and then filtered slowly and gently through a net (mesh size 20 μm). The concentrated tintinnid samples (~150 ml) were fixed with formalin solution to 5% final concentration. Subsamples of 20 ml from well-mixed concentrated samples were pipetted into a sedimentation chamber and settled for 12–24 h [2], and subsequently counted under an Olympus IX 71 inverted microscope (200× or 400×) with photographic measurement system.

Tintinnid species identifications were made on the basis of lorica morphology and dimensions according to literatures [3], [4], [5], [6], [7], [8], [9], [10], [11], [12], [13].

## 2 Data

### 2.1 Tintinnid species richness

**Fig. 1:**
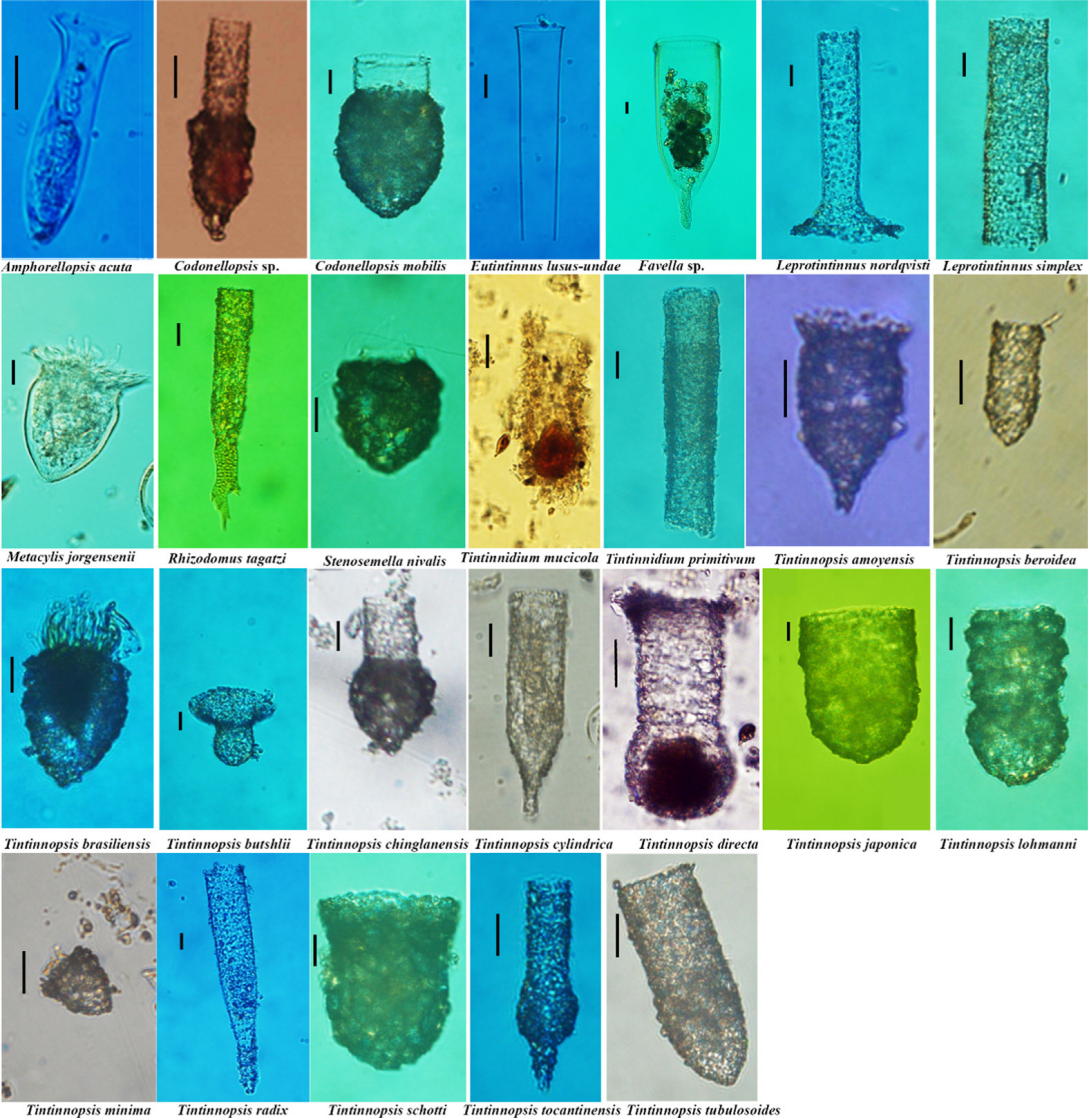
Images of tintinnid species occurred in Jiaozhou Bay during 2003–2012 (scale bar: 20 μm).

A total of 26 species belonging to 9 genera were found in the 428 samples (Fig. 1). Tintinnid species richness ranged from 0 to 20, and the maximum occurred at St. C3 and St. D8 in August 2006. Tintinnid species had been found in all the samples except the sample of St. D8 in December 2009. The ranges of species richness for St. A5, St. C3, St. D8, and St. D7 were 0–16, 0–20, 0–20, and 1–15, respectively. According to the biogeographical pattern of [14], all the 9 genera found belong to neritic (*Favella*, *Leprotintinnus*, *Metacylis*, *Stenosemella*, *Tintinnidium*, *Tintinnopsis*) and cosmopolitan (*Amphorellopsis*, *Codonellopsis*, *Eutintinnus*) biogeographical types.

### 2.2 Tintinnid abundance

The abundance of tintinnid assemblages were different in the four sampling sites (Table 1). The ranges of abundance for St. A5, St. C3, St. D8, and St. D7 were 1–3197 ind L^−1^, 2–1300 ind L^−^^1^, 0–1030 ind L^−^^1^, and 1–1102 ind L^−1^, respectively.

**Table 1:** Data showing tintinnid abundance (ind L^−1^) for each year at each site by month.

Site	Year	Abundance											
		Jan	Feb	Mar	Apr	May	Jun	Jul	Aug	Sep	Oct	Nov	Dec
A5	2003					270	178	46	621	119	49	1452	1
	2004	7	7	12	187	162	3970	181	341	72	149	20	6
	2005	2	11	29	239	297	147	42	229	58	52	45	7
	2006	2	46	127	521	38	39	1355	197	123	56	27	168
	2007	1	5	402	59	235	34	260	421	97	336	263	63
	2008	8	58	45	25	200	74	8	11	81	274	0	0
	2009	23	8	685	60	8	77	1441	528	373	255	20	0
	2010	2	165	79	11	128	21	0	57	4	0	0	45
	2011	0	0	0	175	59	40	829	81	67	51	165	10
	2012	2	4	122	305	0	60	102	100	12	5	385	14
C3	2003					38	740	128	70	42	30	269	18
	2004	27	8	11	57	158	50	150	603	189	15	34	14
	2005	6	7	79	466	624	1084	50	201	82	58	51	24
	2006	35	234	472	59	394	1023	1300	276	91	34	32	92
	2007	4	121	205	15	94	444	22	505	71	31	124	106
	2008	16	63	53	8	22	435	7	67	13	9	0	0
	2009	20	43	490	82	26	23	351	18	32	38	21	0
	2010	21	608	304	39	9	34	0	33	10	0	0	13
	2011	0	0	0	9	96	28	245	186	157	27	4	18
	2012	24	12	63	264	0	28	83	7	11	2	39	60
D7	2003					40	935	54	33	31	11	200	17
	2004	27	4	1	87	11	102	21	1102	94	38	107	8
	2005	20	12	55	577	7	585	83	43	46	13	37	18
	2006	15	91	99	12	32	122	337	293	60	57	18	18
	2007	20	37	147	83	16	38	35	258	23	28	52	64
	2008	30	46	147	8	7	40	4	26	26	22	75	0
	2009	25	8	180	12	4	1	2	12	27	32	32	0
	2010	22	211	0	81	3	28	0	167	11	0	0	11
	2011	0	0	5	3	29	6	122	437	32	9	20	26
	2012	14	10	19	168	0	26	21	196	6	2	4	20
D8	2003					145	675	85	478	28	13	54	33
	2004	10	0	1	32	77	65	39	1030	110	20	55	11
	2005	15	22	12	310	28	578	139	56	46	61	19	17
	2006	11	74	152	16	20	92	347	297	73	36	24	14
	2007	35	20	54	69	13	320	48	278	30	40	75	87
	2008	23	101	91	46	0	769	0	0	29	16	80	0
	2009	9	12	105	80	13	2	10	17	23	16	22	0
	2010	10	181	155	197	11	27	0	47	18	0	0	5
	2011	0	0	0	41	13	2	154	413	66	27	20	23
	2012	10	17	14	77	0	18	57	11	7	5	3	21

Blank means no sampling in the site in the month.

### 2.3 Tintinnid species occurrence

Species occurrence were showed in Table 2, Table 3, Table 4, Table 5 in different site respectively. Based on the multi-year and multi-site investigations, 10 species had a year-round distribution, 2 species could occur in 11 months. Among the 9 genera, genus *Tintinnopsis* had more species (15) than other genera. The remaining genera were represented by 1 or 2 species. All the species have agglutinated lorica except 4 hyaline species: *Amphorellopsis acuta*, *Eutintinnus lusus-undae*, *Favella* sp. and *Metacylis jorgensenii*. All the 4 hyaline species occurred in July-September period. Hyaline forms herein occurred mainly in summer, as well as agglutinated forms peaked in summer.

**Table 2:** Tintinnid species occurrence for each month in each year at St. A5.

Yellow shows the species occurred in the site in the month. sp1 *Amphorellopsis acuta*, sp2 *Codonellopsis* sp., sp3 *C. mobilis*, sp4 *Eutintinnus lusus-undae*, sp5 *Favella* sp., sp6 *Leprotintinnus nordqvisti*, sp7 *L. simplex*, sp8 *Metacylis jorgensenii*, sp9 *Rhizodomus tagatzi*, sp10 *Stenosemella nivalis*, sp11 *Tintinnidium mucicola*, sp12 *Tm. primitivum*, sp13 *Tintinnopsis cylindrica*, sp14 *T. amoyensis*, sp15 *T. beroidea*, sp16 *T. brasiliensis*, sp17 *T. butshlii*, sp18 *T. chinglanensis*, sp19 *T. directa*, sp20 *T. japonica*, sp21 *T. lohmanni*, sp22 *T. minima*, sp23 *T. radix*, sp24 *T. schotti*, sp25 *T. tocantinensis*, sp26 *T. tubulosoides.*

**Table 3:** Tintinnid species occurrence for each month in each year at St. C3.

Yellow shows the species occurred in the site in the month. Sp1, …sp26 see in Table 2.

**Table 4:** Tintinnid species occurrence for each month in each year at St. D8.

Yellow shows the species occurred in the site in the month. Sp1, …sp26 see in Table 2.

**Table 5:** Tintinnid species occurrence for each month in each year at St. D7.

Yellow shows the species occurred in the site in the month. Sp1, …sp26 see in Table 2.
