# Supplementary material for: Dataset of long term variation in species occurrence and abundance of tintinnid assemblages in Jiaozhou Bay, China
Source: Data Brief. 2018 Jun 15;19:1856–64. doi: 10.1016/j.dib.2018.06.010 (PMC6141136; doi:10.1016/j.dib.2018.06.010)
Supplement: Supplementary file 1 — Supplementary material [file mmc1.docx]

Conflict of interest

The authors declared that they have no conflicts of interest to this work.
